# CT scanning for diagnosing blunt ureteral and ureteropelvic junction injuries

**DOI:** 10.1186/1471-2490-8-3

**Published:** 2008-02-07

**Authors:** Sarah J Ortega, Fernado S Netto, Paul Hamilton, Peter Chu, Homer C Tien

**Affiliations:** 1 Trauma Program and Department of Surgery, Sunnybrook Health Sciences Centre, Toronto, Canada; 2 Department of Medical Imaging, Sunnybrook Health Sciences Centre, Toronto, Canada

## Abstract

**Background:**

Blunt ureteral and ureteropelvic (UPJ) injuries are extremely rare and very difficult to diagnose. Many of these injuries are missed by the initial trauma evaluation.

**Methods:**

Trauma registry data was used to identify all blunt trauma patients with ureteral or UPJ injuries, from 1 April 2001 to 30 November 2006. Demographics, injury information and outcomes were determined. Chart review was then performed to record initial clinical and all CT findings.

**Results:**

Eight patients had ureteral or UPJ injuries. Subtle findings such as perinephric stranding and hematomas, and low density retroperitoneal fluid were evident on all initial scans, and prompted delayed excretory scans in 7/8 cases. As a result, ureteral and UPJ injuries were diagnosed immediately for these seven patients. These findings were initially missed in the eighth patient because significant associated visceral findings mandated emergency laparotomy. All ureteral and UPJ injuries have completely healed except for the case with the delay in diagnosis.

**Conclusion:**

Most blunt ureteral and UPJ injuries can be identified if delayed excretory CT scans are performed based on initial CT findings of perinephric stranding and hematomas, or the finding of low density retroperitoneal fluid.

## Background

Ureteral and ureteropelvic junction (UPJ) injuries are rare, and their initial diagnosis is often difficult to make [1-12]. Delays in diagnosis occur in over 50% of cases [3,10]. One reason for delays is that clinical parameters are unreliable for predicting the need to investigate the ureters [4,5,9]. For example, one study reported that none of their patients with ureteral injuries presented with gross hematuria [3]. Furthermore, blunt ureteral injuries are even rarer and more difficult to diagnose [3-6,12]. However, avoiding missed diagnoses is important because studies have linked delays in diagnosis and treatment with poor urologic and overall outcomes [3,4].

CT technology and resolution have improved dramatically over the last decade, and its use for evaluating blunt trauma patients is commonplace. We hypothesize that subtle findings on abdominal CT scanning may suggest the need to further investigate ureteral integrity after blunt trauma, and may reduce the incidence of missed injuries. We describe eight patients at our institution who sustained blunt ureteral injuries, and we report their initial clinical and CT findings.

## Methods

The trauma registry at Sunnybrook Health Sciences Centre, an urban Level I trauma centre in Toronto, Canada was used to identify all blunt trauma patients evaluated from 1 April 2001 to 30 November 2006. The study end-date was selected, as our CT imaging practice changed after November 2006 to require delayed images in all patients with gross hematuria. As well, in September 2002, the trauma fellow at our institution began to present radiological rounds where interesting imaging cases were reviewed. A separate record of all patients with blunt ureteral injuries (and other injuries) was maintained through this review. Patients were included in this study if they had a radiological or intra-operative diagnosis of ureteral or UPJ injury. Patients were excluded if the diagnosis of their genitourinary (GU) tract injury was made at a referring hospital, or if they had sustained either a penetrating or burn injury.

Patient demographics, injury mechanism, Injury Severity Score (ISS), Abbreviated Injury Scale Scores (AIS), length of hospital stay (days), length of intensive care unit stay (days) and in-hospital outcome (dead/alive) were determined from our trauma registry. ISS and AIS were calculated by trauma registry staff after discharge or death.

We then reviewed the hospital chart and radiological record of each study patient; specific attention was focused on initial clinical findings in the trauma room, and initial radiological findings. We also reviewed operative and interventional radiological procedures performed on each study patient, and urologic outcomes.

All abdominal CT scans for trauma during the study period were done with a GE light speed spiral multi-detector scanner [GE Medical Systems, Milwaukee, Wisconsin], using 5 mm collimation. Intravenous contrast was administered at a rate of 3 ml per second for a total of 100–130 ml with a delay of 60 seconds. If an injury to the UPJ or ureter was suspected, delayed (excretory phase) images were obtained. Statistical analysis was performed using SAS software (version 8.02, SAS Institute Inc., Cary, NC). Descriptive statistics are presented (mean ± standard deviation). This study was reviewed and approved by our institutional ethics board.

## Results

During the study period, 4693 blunt patients were evaluated by our trauma service. Of these, 8 patients (0.2%) had a diagnosis of ureteral or UPJ injuries. Five patients were male, and the mean age of study patients was 48 (+/- 16) years. Mean ISS was 45 +/- 16. In five cases, the injury mechanism was motor vehicle collisions; two patients had suffered falls; one patient was a pedestrian struck by an automobile.

Patient characteristics and initial clinical and CT findings are presented in Table I. All eight patients had initial CT scanning performed of the abdomen and pelvis. Patients had multiple clinical findings that warranted CT examination including gross hematuria (7/8), abdominal tenderness (2/8), transient hypotension (2/8) and altered sensorium (4/8). However, none of these patients had overt signs of ureteral or UPJ disruption on initial CT scanning of the abdomen and pelvis. Such signs would include contrast extravasation from the genitourinary tract. Two patients had alternative diagnoses that may have explained the presence of gross hematuria (renal lacerations).

**Table 1:** Patient Characteristics

**Patient No.**	**Age/Gender**	**Mechanism of Injury**	**Hematuria Present (yes/no)**	**Indications for CT Scan of the Abdomen/Pelvis**	**Other Injuries**	**Type of Ureteral Injury**	**Admission CT Findings**	**Delay in Diagnosis**
1	63/M	Car hit by train	Yes	Abdominal pain	Right diaphragm rupture, RLQ hematoma	Left UPJ injury	Stranding and fluid around mid left ureter	No
2	32/F	Motor vehicle collision	Yes	Abdominal pain	C2 fracture, L1-5 fractures, multiple pelvic fractures, right wrist fracture	Right UPJ injury	Low density fluid collection around the kidney/Proximal ureter	No
3	55/M	Fall from 25 ft	Yes	Transient hypotension	Liver lacerations, renal lacerations, multiple rib fracture, R pneumothorax, multiple pelvic fractures, L2 fracture	Mid right ureteral injury	Low density fluid collection along right ureter	No
4	46/M	Car struck by a train	Yes	Unreliable clinical examination due to intubation and sedation	Bilateral renal lacerations, right femoral fracture	Proximal right ureteral injury	Mild degree of fluid and inflammatory change in perinephric fat	No
5	21/F	Motor vehicle collision	Yes	Unreliable clinical examination due to intubation and sedation	Right liver laceration, pancreatic contusion, pelvic fractures. L3/4 fractures	Proximal left ureteral injury	Fluid collection in the perinephric space	No
6	69/M	Pedestrian struck by a car	Yes	Transient episode of hypotension	Splenic laceration, multiple rib fractures, pelvic fracture, bilateral pneumothoraces	Left UPJ injury	Left perinephric hematoma	No
7	42/M	Fall from 100 ft	Yes	Unreliable clinical examination from complete paraplegia with sensory deficit	Comminuted L2/3 fractures	Right UPJ injury	Perinephric hematoma	No
8	56/F	Head-on motor-vehicle collision	No	Unreliable clinical examination due to intubation and sedation	Right kidney lacerations, liver and pancreatic contusion, right adrenal hematoma	Mid right ureteral injury	Small amount of low density fluid adjacent to the right kidney	Yes

**Figure 1:**
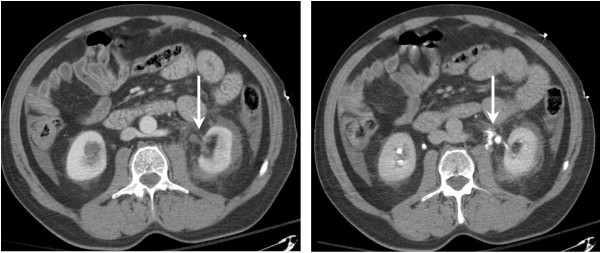
69 year old male. Contrast enhanced (left) and delayed (right) CT scans demonstrating low density fluid around the left kidney and ureter, and contrast extravasation from the proximal left ureter respectively.

**Figure 2:**
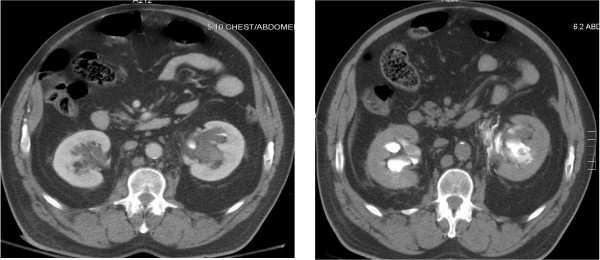
63 year old male. Contrast enhanced (left) and delayed (right) CT scans demonstrating stranding and fluid around the mid left ureter, and a left UPJ injury respectively.

In all eight cases, however, there were subtle findings on initial CT scanning that suggested ureteral or UPJ injury. These included perinephric stranding, low density fluid around the kidney and ureters, and perinephric hematomas (see Table 1). In seven of the eight cases, these findings were identified immediately and prompted a repeat CT scan to obtain delayed excretory images of the kidneys and ureters. Overt signs of ureteral or UPJ injuries (GU contrast extravasation) were then evident in all seven cases (See Figures 1 and 2).

In the eighth case, the initial findings on CT scanning were originally missed, but evident on retrospective review. This was a case of a 56 year old woman who sustained injuries from a motor vehicle collision. Her initial abdominal CT findings consisted of right sided kidney lacerations to the upper and lower poles with some surrounding low density fluid adjacent to the kidney, retroperitoneal free air with associated duodenal thickening, liver and pancreatic contusions, right adrenal and right diaphragmatic hematomas, and a contained inferior vena caval injury. The initial CT interpretation focused on her other significant intra-abdominal and retroperitoneal injuries. She subsequently went to the operating room for a laparotomy, and her urological injuries only became evident on post-operative day number five. She began complaining of a significant amount of abdominal pain, and repeat CT scan of the abdomen and pelvis showed mild hydronephrosis with a new right-sided retroperitoneal fluid collection. Delayed imaging then revealed urological contrast extravasation consistent with a mid ureteral injury. There was contrast in the distal ureter, however, indicating a partial injury.

No patients died in this series. All seven patients who had their ureteral injuries diagnosed immediately were successfully treated with non-operative management, and all healed completely. The patient with a delayed diagnosis was treated with a right nephro-ureterostomy tube, but the ureteral injury has not yet completely healed.

## Discussion

Most ureteral or UPJ injuries due to trauma are the result of penetrating injury; blunt injuries are extremely rare. There is substantial variation in practice patterns for diagnosing these injuries. Some centres used gross hematuria as the clinical trigger to conduct genitourinary tract (GU) investigations. In these reports, retrograde urography and intravenous pyelography have been used to make the diagnosis [3]. Other centres have found that CT scanning is useful in making the diagnosis of ureteral or UPJ injuries by showing contrast extravasation from the GU tract [1-3,5]. All previous studies have reported a high frequency of missed UPJ and ureteral injuries. One of the reasons for missing this injury is that focused investigations of the GU tract (IVP, CT scanning with delayed images) were being performed based on initial clinical parameters. However, no set of initial clinical parameters have been found to reliably predict UPJ or ureteral injuries [4,5,9].

In this report, we report our experience with eight patients who suffered blunt UPJ or ureteral injuries. In all eight cases, initial CT scanning of the abdomen and pelvis was performed for a variety of clinical indications, although none of the indications were present in all patients. Indications included gross hematuria, abdominal or flank pain, altered sensorium and transient hypotension. Initial CT scanning was not diagnostic for GU tract injuries in any cases. However, subtle GU findings were evident in all eight patients. These findings included mild perinephric stranding, low density fluid around the kidney and ureter, and perinephric hematoma. In seven patients, these findings were noted on the initial CT report, and prompted delayed CT imaging which showed overt contrast extravasation from the UPJ or ureters. For one patient, these initial CT findings were missed, resulting in a delay in diagnosis. One factor which may have contributed to this delay was the presence of other major visceral injuries on CT scanning that required emergency operative intervention. Another contributing factor may be lack of experience in the reporting radiology or trauma fellows in recognizing the significance of these subtle findings.

## Conclusion

In this small retrospective review, we found that the initial CT findings of mild perinephric stranding, low density retroperitoneal fluid around the GU tract and perinephric hematomas should prompt the evaluation of the kidneys and ureters by delayed excretory scanning. Clinicians should be diligent in reviewing the initial CT scan for all abnormal findings, even in the presence of other serious injuries that require emergency surgery; otherwise, delays in diagnosing GU tract injuries will persist. Further studies are required to determine the sensitivity and specificity of these findings for GU tract injuries.

## Competing interests

The author(s) declare that they have no competing interests.

## Authors' contributions

SJO and FSN reviewed the charts of all study patients. PH reviewed the imaging of all study patients. PC and HCT developed the concept for this project, and HCT provided methodological advice. SJO wrote the manuscript. All authors have read and approved the final manuscript.

## Pre-publication history

The pre-publication history for this paper can be accessed here:


